# Effects of Lebanese Folk Herbs on Adult Male Rats: Hepatic and Renal Toxicity, Histological, and Biochemical Studies

**DOI:** 10.3390/nu17050875

**Published:** 2025-02-28

**Authors:** Rana R. Khalaf, Noura S. Abouzeinab, Mahmoud I. Khalil

**Affiliations:** 1 Department of Biological Sciences, Faculty of Science, Beirut Arab University, P.O. Box 115020, Riad El Solh, Beirut 11072809, Lebanon; rrk297@student.bau.edu.lb (R.R.K.); or mahmoud_ibrahim@alexu.edu.eg (M.I.K.); 2 Molecular Biology Unit, Department of Zoology, Faculty of Science, Alexandria University, Alexandria 21500, Egypt

**Keywords:** folk herbs, toxicity, liver, kidney, oxidative stress, histology

## Abstract

**Background/Objectives:** *Lepidium sativum*, *Ferula hermonis*, *Origanum majorana*, and *Eruca sativa* are frequently consumed as a traditional Middle Eastern medicine to promote health and treat various diseases. However, concerns have been raised about their possible harmful effect in humans. Limited research has examined their chronic toxicity in rats, and their combined exposure effects are still unknown. Hence, this research aimed to evaluate their potential hepato- and nephrotoxic effects. **Methods:** Aqueous extracts of the selected plants, with a dose of 100 mg/kg, were administered separately and as a mixture daily for 60 days. Blood and tissue were sampled from 28 rats, and organ weight, biochemical markers for kidney and liver function, and histopathological examination were assessed. **Results:** The results indicated increased liver weight, liver and kidney malondialdehyde, alanine transaminase, and urea, and decreased serum creatinine and kidney glutathione levels. Additionally, histological examinations showed liver and kidney architectural damage. Further, the extent of toxicity varied among the plants. **Conclusions:** In conclusion, the results revealed that the corresponding plant extracts’ oral administration affects biological functions and promotes liver and kidney oxidative damage in rats, with FH and ES exhibiting the highest level of liver toxicity and ES and MIX showing the highest level of kidney toxicity.

## 1. Introduction

Historically, medications have been primarily sourced from plants. Due to their high therapeutic potential and ability to improve well-being, which has improved quality of life since ancient times, herbal drugs are widely accepted by the general population [1]. The main advantages of herbal medicines include relatively less pharmacological side effects and cost-effectiveness [2].

*Lepidium sativum* (LS), *Eruca sativa* (ES), *Ferula hermonis* (FH), and *Origanum majorana* (OM) are plants that all originate from the Mediterranean region and are commonly used [3,4,5,6]. In fact, in both in vitro and in vivo investigations, LS, or garden cress, and its extracts, along with ES (rocket) extracts, have shown to exhibit a varied array of biological activities, including antibacterial, antioxidant, and anticancer properties [6,7,8,9]. OM, also known as sweet marjoram, has been used in folkloric medicine to treat a wide range of illnesses, including rheumatologic, neurological, and respiratory ailments [5,10,11]. FH, or Lebanese Viagra, has been conventionally consumed as an aphrodisiac and to treat skin infections, stomach problems, and neurological conditions [12]. These plants are also reputed to enhance fertility parameters [13,14,15,16].

The liver is an organ involved in different tasks including detoxification, metabolism, digestion, supporting immunity, in addition to vitamin storage. The liver’s capability to function properly might be influenced by food or medication misapplication [17]. Natural products used in folkloric medicine can cause herb-induced liver injury and hepatotoxicity [18]. For example, *Polygonum multiflorum* Thunb is toxic to the liver, and its hepatotoxicity can reach different degrees and can even be fatal [19]. *Teucrium chamaedry*, also known as germander and consumed for weight loss, can cause hepatotoxicity and hepatitis [20]. In addition, pyrrolizidine alkaloid (PA)-induced hepatic sinusoidal obstruction syndrome cases resulting from the consumption of PA-containing *Gynura segetum* were reported by Lin et al. [21].

The kidneys control various bodily processes, including intra- and extracellular volume status and acid–base balance, in addition to facilitating the excretion of uremic poisons through the urine [22]. In herbal medicine, more than 100 different types of herbs have been demonstrated to be toxic to the kidneys, yet their exact toxic constituents are still unknown [23]. An example is *Rheum palmatum*, which was found to trigger an inflammatory response, activate the production of caspase-3, and induce cell apoptosis and oxidative stress with an imbalance in the glutathione antioxidant system in the kidneys of mice [23].

Recently, the advantageous effect of the currently chosen folk herbs on spermatogenesis in rats and the impact of their corresponding aqueous extracts on male Sprague Dawley (SD) rats’ fertility were investigated. However, little is known regarding their possible toxic impact on other organs, specifically the liver and kidneys. Consequently, the purpose of this investigation was to explore the individual and combined effects of LS, OM, FH, and ES on liver and renal toxicity in SD rats, following a 60-day treatment via oral gavage. Toxicity assessments involved histopathological examinations, biochemical analyses, and evaluation of oxidative stress markers.

## 2. Materials and Methods

### 2.1. Collection and Preparation of Extract

*Lepidium sativum* seeds, *Ferula hermonis* roots, shade-dried leaves of *Origanum majorana*, and *Eruca sativa*’s overground parts were acquired from a regional market. Following storage in the dark, plant material was identified at Beirut Arab University’s Herbal Garden for Lebanese Medicinal and Aromatic Plants in the Research Center for Environment and Development (RCED), Bekaa Campus, Lebanon (BAU Herbal Garden for Lebanese Medicinal and Aromatic Plants, Beirut Arab University, accessed on 10 September 2022). The leaves and stems of *Eruca sativa* were cut into thin pieces after being shade-dried. A mill was used to grind the corresponding plant parts. An amount of 500 mL of distilled water was combined with 25 g of each plant to prepare the water extract. After that, the solution was heated to 95 °C in a water bath for 30 min. Following extraction, vacuum filtration was performed through Büchner funnel filtration with adequate filter papers. The obtained solution was aliquoted and kept at −4 °C until needed. Vitamin E (VitE) was obtained from *Webber Naturals*^®^, Coquitlam, BC, Canada).

### 2.2. Animals

Twenty-eight mature male Sprague Dawley rats (*Rattus norvegicus*) (aged 6 to 7 weeks) in good health, with an average body weight of 180 ± 5 g, were sourced from the animal house facility at Beirut Arab University. The optimal animal sample size needed to obtain a strong statistical power while preserving ethical considerations was taken into account when choosing the sample size. The animals were kept in a well-ventilated environment (temperature 23 ± 2 °C and humidity 60–70%); photoperiod: 12 h of natural light and 12 h dark. Standard commercial pellet food and tap water were available for animals ad libitum. All laboratory animals were treated humanely and monitored carefully throughout the study according to the guidelines provided by the National Institutes of Health (NIH) and to prevent any discomfort or pain from exceeding the threshold set by Beirut Arab University’s ethical committee, the Institutional Review Board (IRB) (protocol number: 2023-A-0050-S-P-0513).

### 2.3. Experimental Design

This is a follow-up of our previous investigation where the same experimental design was applied [24]. After the adaptation period, the rats were randomized into 7 groups (4 rats per group) (Table 1) and received a daily treatment via oral gavage for sixty days, as follows: Group “Ctrl”: control group administered normal saline (NaCl) in a volume that was equivalent to what the experimental groups received. Group “LS”: received aqueous extract of *Lepidium sativum*. Group “OM”: received aqueous extract of *Origanum majorana*. Group “FH”: received aqueous extract of *Ferula hermonis*. Group “ES”: received aqueous extract of *Eruca sativa*. Group “Mix”: received a mix of the afore-mentioned plant extracts. Group “VitE”: received vitamin E as a positive control (known for its antioxidant, anti-inflammatory, and protective role [25]). All treatments were given at a dose equal to 100 mg/kg, which was determined based on prior studies [8,26,27,28,29]. The animals were inspected every other day for any sign of toxicity, their body weight was monitored daily during the trial, and the rats’ daily food and water intake was tracked. The experimental animals were sacrificed on day sixty, and the weight of the kidneys and livers was noted.

To prevent any bias, animal care, collection of samples, and data analysis were all carried out in a blinded form, with researchers unaware of assignments of groups at any stage. After data collection and analysis, blinding was removed. No experimental units, animals, nor any data point were excluded from the study during any stage since the selected exposure doses did not cause any pain or signs of mortality or any adverse effects in the animals.

### 2.4. Sample Collection

Rats were sacrificed after 60 days of treatment, and blood samples were collected via heart puncture and then centrifuged. Kidney and liver samples were excised and weighed to determine liver and kidney weights and indices. The organ weight was normalized to body weight by dividing and then multiplied by 100 to calculate the organ index. Part of the kidney and liver samples were stored in 10% formalin fixative for histological examination. The remaining parts were kept at −80 °C to prepare the corresponding tissue homogenates for further biochemical investigation.

### 2.5. Biochemical Analysis

Serum urea and creatinine concentrations, which serve as markers of renal excretory function, were measured, as were the activities of enzyme markers of hepatocellular damage, such as alanine transaminase (ALT) and aspartate aminotransferase (AST). Commercially available kits were used for all analyses (ALTL, ASTL, UREAL, CREJ2, Roche, Munich, Germany) based on the spectrophotometric method (Cobas c501, Roche Diagnostics, Germany), according to the manuals supplied.

### 2.6. Liver and Kidney Lipid Peroxidation and Antioxidant Enzyme

Liver and kidney tissue parts were homogenized using a protease inhibitor cocktail III-containing 0.1 M sodium phosphate buffer (PH 7.4). Centrifugation was then performed for 15 min at 10,000 rpm to separate the supernatant, which was later utilized to evaluate the antioxidant as well as lipid peroxidation markers.

#### 2.6.1. Superoxide Dismutase (SOD)

Measurement of superoxide dismutase enzyme activity in the liver and kidneys was performed based on previously described methods [30]. Superoxide free radicals were produced in vitro during this reaction in the presence of strong light. The superoxide radical transforms the yellow chemical nitro blue tetrazolium (NBT) into the blue monoformazon. The competitive suppression of NBT reduction by the superoxide radical is used to measure SOD activity. The absorbance was assessed spectrophotometrically at 560 nm (Thermo Evolution60, Thermo Fisher Scientific Inc., Waltham, MA, USA).

#### 2.6.2. Reduced Glutathione (GSH)

The glutathione level in the sample was precisely determined using the technique published by Moron et al. [31]. Briefly, a yellow derivative is produced when 5,5′-dithio-bis (2-nitrobenzoic acid) (DTNB) oxidizes glutathione, where the absorbance is measured at 405 nm using a microplate reader (Multiskan™ FC Thermo Fisher Scientific Inc., Waltham, MA, USA),

#### 2.6.3. Lipid Peroxidation

Following the method of Heath and Packer [32], the level of MDA was quantified to estimate lipid peroxidation. A pink molecule is produced following addition of thiobarbituric acid (TBA) to the sample. This molecule is spectrophotometrically absorbed at 535 nm using a microplate reader (MultiskanTM FC Thermo Fisher Scientific Inc., Waltham, MA, USA).

### 2.7. Histological Observation

For histological examination, livers and kidneys were fixed in formalin fixative. After fixation, the tissue sections were dehydrated in ethanol solutions with increasing concentrations, cleared with xylene, and embedded with paraffin. A rotary microtome (Leica RM2235, Leica Microsystems, Deerfield, IL, USA) was employed for sectioning. Thin 5 µm sections were subsequently stained with Harris hematoxylin and eosin (H&E) stain and inspected via a bright field microscope (Leica DM500 with ICC50 W CAM, Leica Microsystems, Deerfield, IL, USA). In the liver, cytoplasmic vacuolization, damaged or necrotic hepatocytes, inflammatory cell infiltration, pyknosis, bile duct proliferation, and Kupffer cells hyperplasia were among the criteria used in the histopathological scoring of the average tissue injury score. In contrast, the kidneys’ necrotic tubules, brush border loss, glomerular atrophy, and tubular degeneration and vacuolization were assessed. The extent of organ injury was semi-quantified using a 5-point scale (0–5), where 0 = normal, 1 = mild, 2 = moderate, 3 = moderate to severe, 4 = severe, and 5 = highly severe, as previously described by scientists [33,34].

### 2.8. Statistical Analysis

Differences among groups were analyzed statistically by performing one-way analysis of variance (ANOVA) using SPSS version 24. Data were reported as means ± SEM. Statistical differences with a *p* < 0.05 were considered significant.

## 3. Results

### 3.1. Total Body Weight, Organ Weight, and Organs Indices

At the end of the experiment, body weights were reported as shown in Table 2. The BW of the treated groups did not considerably differ from the control group after the administration of plant extracts. Similarly, the plant extracts showed no major change in kidney weight and kidney index (*p* > 0.05). On the contrary, ES, LS, and VitE increased the liver weight and index of animals significantly, in comparison with the Ctrl group.

### 3.2. Serum Hepatic and Renal Functional Markers

**Figure 1:**
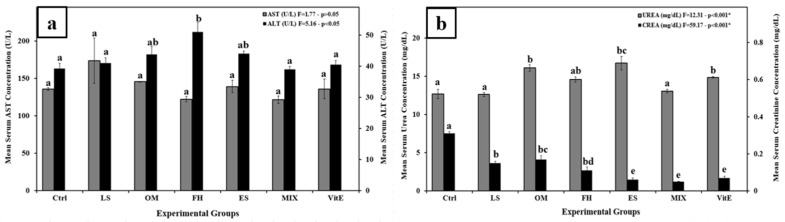
Changes in (**a**) AST (U/L) and ALT (U/L) among experimental groups. (**b**) Serum urea (mg/dL) and CREA (mg/dL) among experimental groups. Different letters indicate significance between groups (*p* < 0.05, one-way ANOVA followed by Tukey’s post-hoc test; * indicates high significance at *p* < 0.001). Ctrl, control group; FH, *Ferula hermonis*-treated group; OM, *Origanum majorana*-treated group; ES, *Eruca sativa*-treated group; LS, *Lepidium sativum*-treated group; Mix, plant mixture-treated group; VitE, vitamin E-treated group. Data are means ± SEM; n = 4 rats per group. The error bar indicates the standard error of the mean (SEM).

The change in AST and ALT levels, in all plant-treated animals, remained insignificant relative to the Ctrl group, with the exception of the FH group, where the ALT level increased significantly when compared to Ctrl (Figure 1a). Furthermore, urea levels were significantly elevated with OM, ES, and VitE treatments compared to the control. However, compared to Ctrl, creatinine levels significantly dropped across all extract-treated groups.

### 3.3. Changes in Lipid Peroxidation and Antioxidant Markers

**Figure 2:**
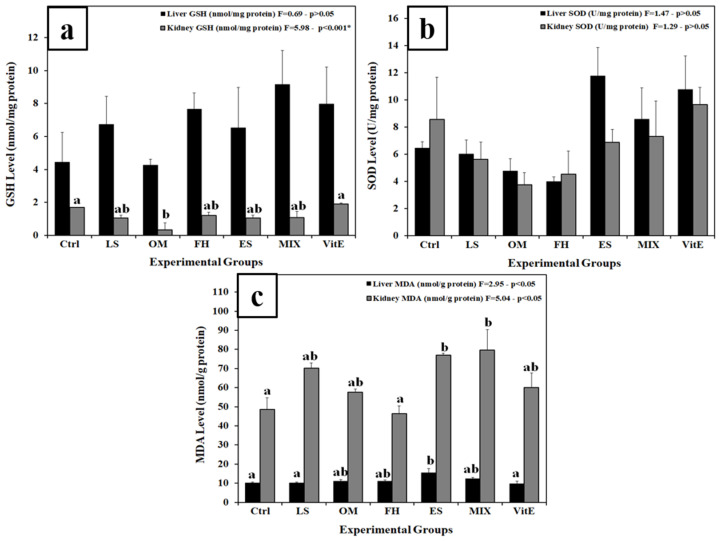
Changes in (**a**) liver and kidney GSH (mmol/mg protein), (**b**) liver and kidney SOD (U/mg protein), and (**c**) liver and kidney MDA (mmol/g protein) among experimental groups. Different letters indicate significance between groups (*p* < 0.05, one-way ANOVA followed by Tukey’s post-hoc test; * indicates high significance at *p* < 0.001). Ctrl, control group; FH, *Ferula hermonis*-treated group; OM, *Origanum majorana*-treated group; ES, *Eruca sativa*-treated group; LS, *Lepidium sativum*-treated group; Mix, plant mixture-treated group; VitE, vitamin E-treated group. Data are means ± SEM; n = 4 rats per group. The error bar indicates the standard error of the mean (SEM).

The levels of liver GSH in all experimental groups did not statistically differ compared to the control. On the other hand, OM significantly decreased kidney GSH levels compared to Ctrl (*p* < 0.001), while the remaining groups were insignificantly different from the control regarding kidney GSH levels (Figure 2a). Plant treatment in all experimental groups did not, statistically, affect the SOD levels in both organs compared to the basal value (Ctrl) (Figure 2b). Liver MDA was significantly increased in rats treated with ES, in comparison with Ctrl, whereas kidney MDA levels were highly upregulated in the animal groups receiving ES and Mix treatments, relative to the control (*p* < 0.01). No significant changes were recorded in the remaining groups (Figure 2c).

### 3.4. Histopathological Findings

**Figure 3:**
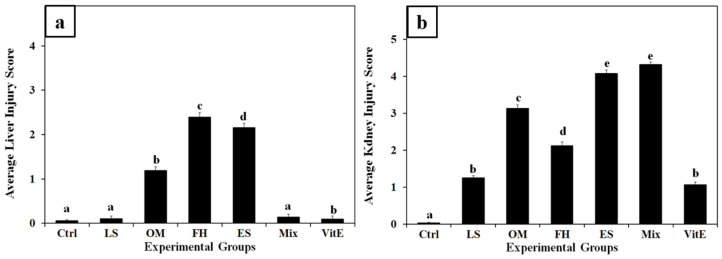
Average (**a**) liver injury score and (**b**) kidney injury score in experimental groups. Different letters indicate significance among groups (*p* < 0.05, one-way ANOVA followed by Tukey’s post-hoc test). Ctrl, control group; FH, *Ferula hermonis*-treated group; OM, *Origanum majorana*-treated group; ES, *Eruca sativa*-treated group; LS, *Lepidium sativum*-treated group; Mix, plant mixture-treated group; VitE, vitamin E-treated group. Data are means ± SEM. The error bar indicates the standard error of the mean (SEM).

The effects of the toxicities of the folk herbs used were examined. Most organs, including the liver and kidneys, when compared to controls, did not show obvious gross pathological modifications. The liver and kidneys’ H&E-stained histological slides were compared to the controls and semi-quantitatively evaluated for histological changes. Figure 3 displays the scoring of histological damage and modification, indicating the average severity of liver and kidney injury among treatment groups.

**Figure 4:**
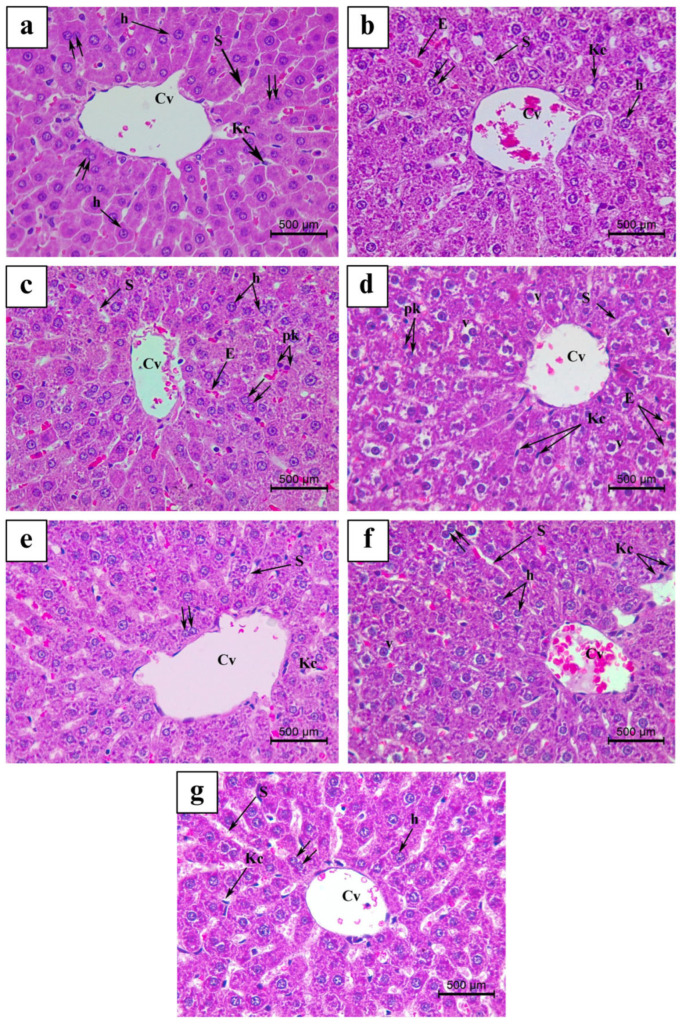
Light micrograph representing H&E-stained liver tissue at pericentral area of the following: (**a**) Ctrl, control group; (**b**) LS group (*Lepidium sativum*-treated group); (**c**) OM group (*Origanum majorana*-treated group); (**d**) FH group (*Ferula hermonis*-treated group); (**e**) ES group (*Eruca sativa*-treated group); (**f**) Mix group (plant mixture-treated group); (**g**) positive control, VitE group (vitamin E-treated group). (Magnification: ×400; scale bar: 500 μm). Cv: central vein; Kc: Kupffer cell; S: sinusoid; h: hepatocyte; E: erythrocyte; pk: pyknotic nuclei; v: vacuolization; double arrow: bi-nucleated hepatocyte.

**Figure 5:**
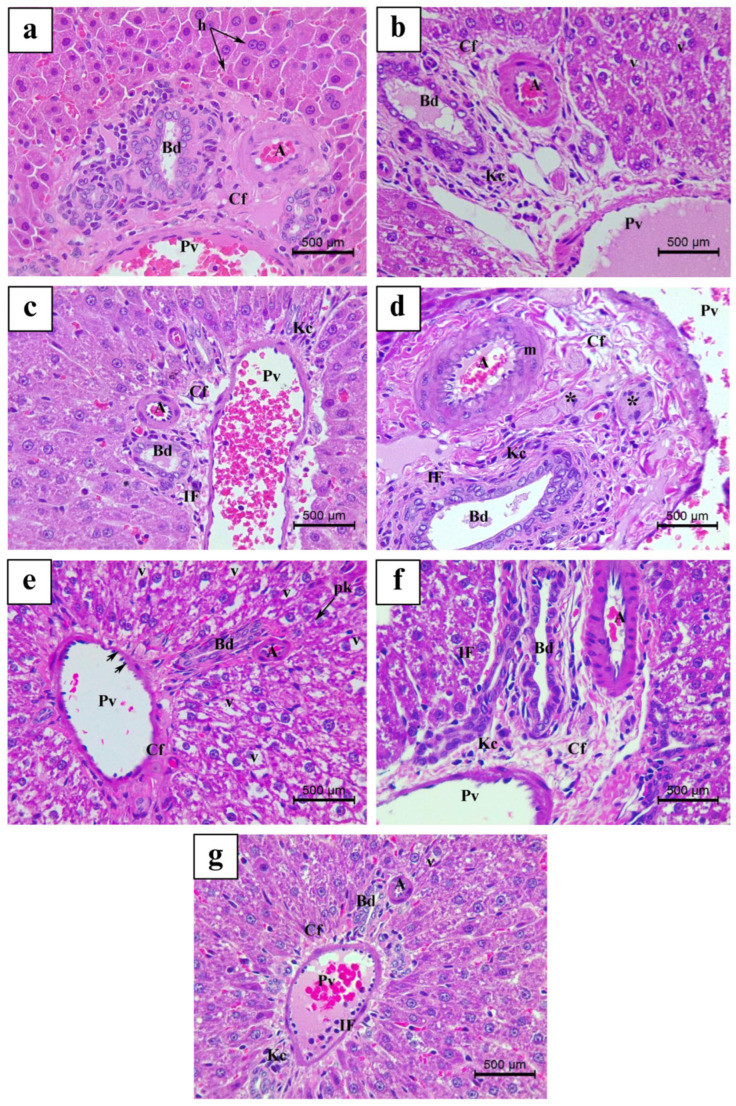
Light micrograph representing H&E-stained liver tissue at pericentral area of the following: (**a**) Ctrl, control group; (**b**) LS group (*Lepidium sativum*-treated group); (**c**) OM group (*Origanum majorana*-treated group); (**d**) FH group (*Ferula hermonis*-treated group); (**e**) ES group (*Eruca sativa*-treated group); (**f**) Mix group (plant mixture-treated group); (**g**) positive control, VitE group (vitamin E-treated group). (Magnification: ×400; scale bar: 500 μm). Pv: portal vein; Bd: bile duct; A: artery; m: muscular layer; Cf: connective tissue fiber; Kc: Kupffer cell; S: sinusoid; h: hepatocyte; E: erythrocyte; pk: pyknotic nuclei; v: cytoplasmic vacuolization; double arrow: bi-nucleated hepatocyte; arrowhead: elongation of epithelial lining; *: Kupffer cell hyperplasia; IF: inflammatory cell infiltration.

Analysis of H&E-stained liver sections from the Ctrl group is represented in Figure 4a and Figure 5a. Besides, histological examination of LS-, Mix-, and VitE-treated liver sections revealed normal hepatic architecture in the pericentral and periportal areas (Figure 4b,f,g and Figure 5b,f,g). However, H&E-stained histological slides of OM- as well as FH- and ES-treated livers exhibited mild to moderate histopathological alterations, respectively (Figure 4c–e and Figure 5c–e). The histological observation and analysis under light microscopy for liver sections receiving OM treatment showed mild morphological changes in the liver architecture relative to the Ctrl group not receiving any plant treatment (Figure 3, Figure 4c and Figure 5c); it showed improper trabecular arrangement of hepatocytes, since most of the hepatocytes appeared irregularly radiating from the slightly congested central vein. Most hepatocytes exhibited a normal histological appearance, whereas few of them had densely stained pyknotic nuclei in specific and limited foci. The hepatic sinusoids appeared mildly dilated and congested with few erythrocytes. Furthermore, the portal area (Figure 5c) showed a congested portal vein accompanied by a minimal incidence of inflammatory cellular infiltration as well as fibrosis. In addition, aggregates of Kupffer cells were mildly scattered within the portal area.

The liver tissues, under the influence of FH treatment, showed mild to severe morphological alterations, which were more severe than those described in the OM-treated group (Figure 4d and Figure 5d). Surprisingly, after FH treatment, significant cellular degenerative alterations and moderate to severe hepatic histopathological abnormalities were evidenced, when compared to the Ctrl group (Figure 3). These modifications comprise a severe loss of hepatic trabecular architecture, where most hepatocytes appeared ballooned and revealed severe fatty alterations including signs of micro-steatosis (Figure 4d) with great cytoplasmic vacuolization. At some hepatic necrosis foci, densely stained pyknotic nuclei were frequently found (Figure 4d). Besides, the portal area displayed necrotic foci among the hepatocytes, a proliferating bile duct, and a dilated and congested portal vein with moderate fibrosis manifested by increased network of connective tissue fibers. Furthermore, periportal hepatocytes were shown to have necrotic foci, which were supplemented by noticeable exudation invasion and masses of inflammatory cell infiltration between the portal area and surrounding the portal vein (periportal inflammatory cellular infiltration). Kupffer cell activation was highly detected, where hyperplasia was formed by highly clustered and aggregated Kupffer cells (histiocytosis) (Figure 4d and Figure 5d). In addition, the hepatic artery appeared severely congested with a thickened muscular layer (Figure 4d and Figure 5d).

However, ES treatment displayed histological changes in H&E-stained liver tissues, similar to those described in FH, and were graded moderate with less severity (Figure 3). The pericentral area demonstrated normal liver architecture with regular hepatic trabecular arrangement. However, the portal area exhibited severe histopathological alteration compared to the Ctrl group. The endothelial lining of the portal vein showed signs of excessive elongation, making it appear dilated, congested, and associated with moderate fibrosis (Figure 5e). Most periportal hepatocytes were hypertrophied and exhibited high levels of ballooning and vacuolization of the cytoplasm, thus revealing severe signs of micro-steatosis. Pyknotic nuclei were densely stained and were detected at minimal foci (Figure 5e).

**Figure 6:**
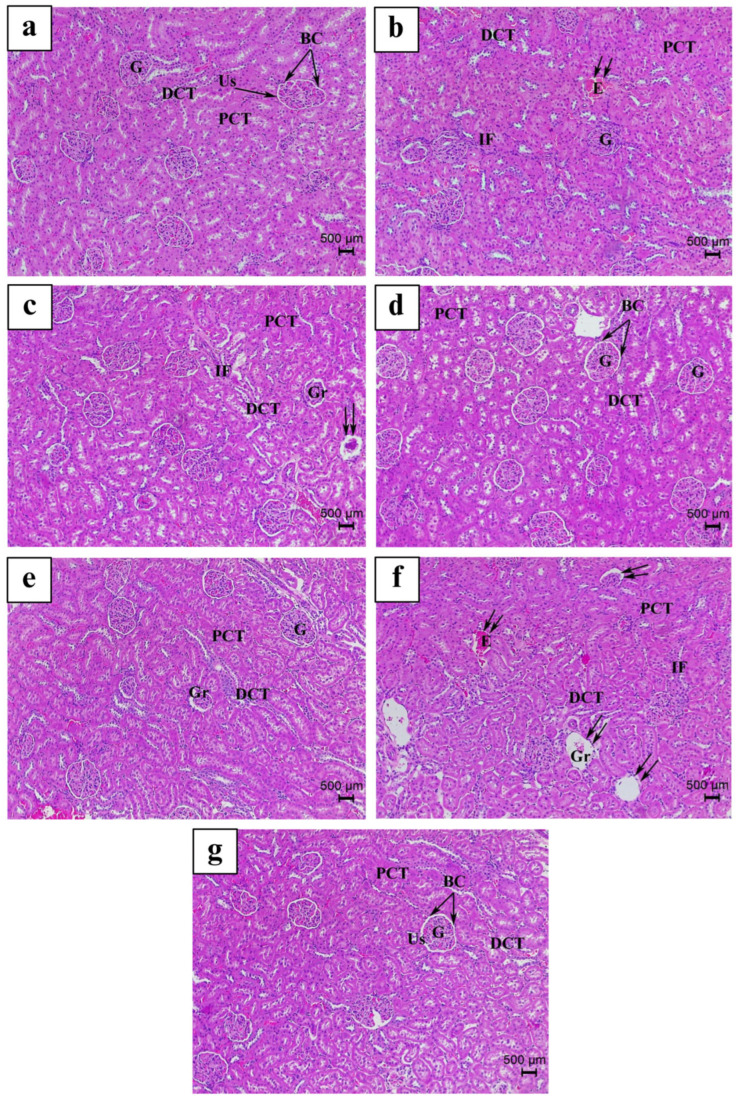
Light micrograph representing H&E-stained kidney tissue of the following: (**a**) Ctrl, control group; (**b**) LS group (*Lepidium sativum*-treated group); (**c**) OM group (*Origanum majorana*-treated group); (**d**) FH group (*Ferula hermonis*-treated group); (**e**) ES group (*Eruca sativa*-treated group); (**f**) Mix group (plant mixture-treated group); (**g**) positive control, VitE group (vitamin E-treated group). (Magnification: ×100; scale bar: 500 μm). BC: Bowman’s capsule; G: glomerulus; Gr: reduced glomerulus; DCT: distal convoluted tubule; Us: urinary space; PCT: proximal convoluted tubule; double arrow: atrophied renal corpuscle with Gr; E: congestion with erythrocytes; IF: peritubular infiltration.

**Figure 7:**
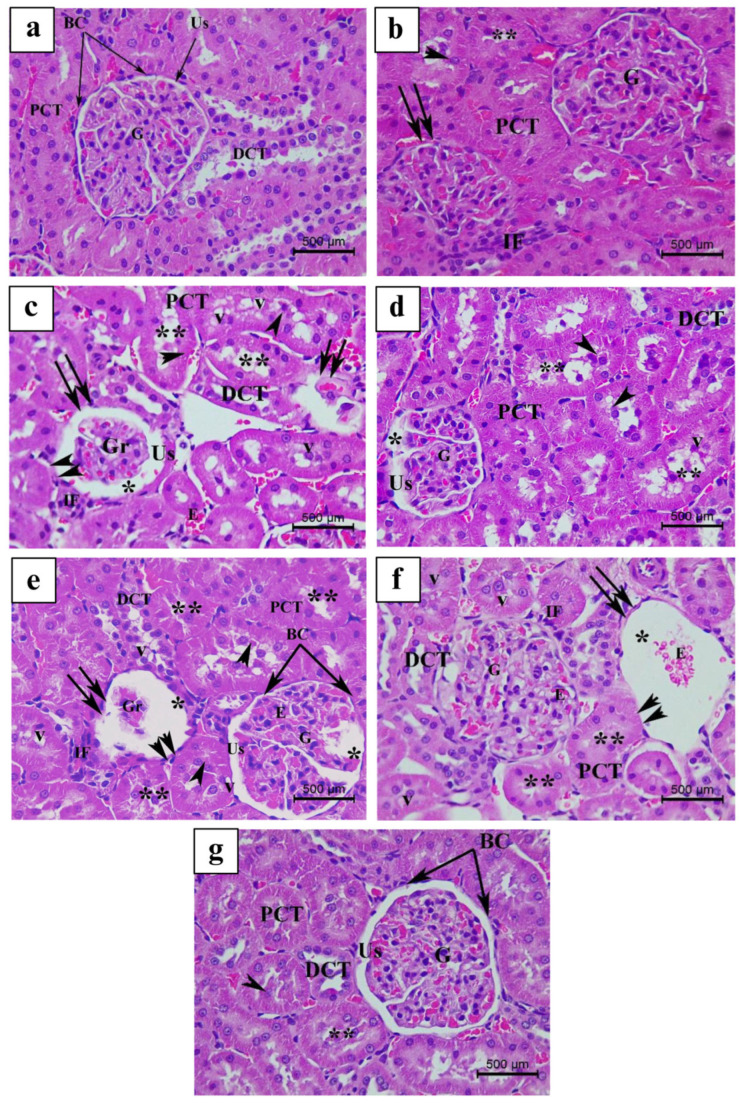
Light micrograph representing H&E-stained kidney tissue of the following: (**a**) Ctrl, control group; (**b**) LS group (*Lepidium sativum*-treated group); (**c**) OM group (*Origanum majorana*-treated group); (**d**) FH group (*Ferula hermonis*-treated group); (**e**) ES group (*Eruca sativa*-treated group); (**f**) Mix group (plant mixture-treated group); (**g**) positive control, VitE group (vitamin E-treated group). (Magnification: ×400; scale bar: 500 μm). BC: Bowman’s capsule; G: glomerulus; Gr: reduced glomerulus; DCT: distal convoluted tubule; Us: urinary space; PCT: proximal convoluted tubule; double arrow: atrophied renal corpuscle with Gr; E: congestion with erythrocytes; *: dilated Us; **: loss of PCT brush borders; v: cellular vacuolization cell; IF: peritubular infiltration; arrowhead: damaged PCT with cellular shedding; double arrowhead: desquamated lining of BC.

The control groups’ kidney tissues are presented in (Figure 6a and Figure 7a). When comparing with the untreated Ctrl animals, the oral treatment of 100 mg/kg LS, VitE, and FH, respectively (Figure 6b,d,g and Figure 7b,d,g), demonstrated modest to moderate renal pathological alterations (Figure 3). The most common and distinguishable pathological features in both groups were the following: reduction of renal corpuscle dimensions with mild dilatation of the urinary space, mild infiltration of peritubular inflammatory cells with few convoluted signs of congestion, few proximal convoluted tubules (PCTs) exhibited a mild loss of brush border with slight PCT cellular displacement and shedding toward the lumen (Figure 6b,d,g and Figure 7b,d,g). On the other hand, OM as well as ES and Mix treatments (Figure 6c,e,f and Figure 7c,e,f), exhibited additional renal histopathological features compared to Ctrl as well as LS and FH groups. These variations were graded as moderate–severe and severe, respectively (Figure 3). Light microscopical inspection of H&E-stained kidney sections from the OM group revealed a moderate reduction in the dimensions of some renal corpuscles with reduced and congested glomeruli. Besides, some Bowman’s capsules (BCs) appeared desquamated at certain foci enclosing a moderate dilatation of the urinary space (Figure 6c and Figure 7c). In addition, both PCTs and distal convoluted tubules (DCTs) exhibited moderate tubular degenerative and necrotic features. Some PCTs revealed a noticeable loss of brush borders with a considerable amount of cellular shedding, where some PCT cells attained a relative cytoplasmic vacuolization and densely pyknotic nuclei. Few DCTs attained irregular tubular arrangement, and moderate inflammatory cellular infiltration and congestion were observed among some PCTs and DCTs (Figure 7c).

On the other hand, ES and Mix oral administration exhibited significant alterations in the architecture and structures of the renal cortex compared to the Ctrl group as well as the rest of the treated groups. Histological examination of H&E-stained ES and Mix (Figure 3, Figure 6e,f and Figure 7e,f) groups displayed severe atrophy of a remarkable number of renal corpuscles with signs of glomerular necrosis, where most glomeruli appeared highly reduced, shrunk, and congested. In addition, some glomeruli exhibited a severe atrophied appearance with a complete degeneration and loss of glomerular cells. Besides, BC showed an extensive desquamation of its epithelium at several foci, accompanied with a wide and severe dilatation of the urinary space. Moreover, a severe distortion of renal tubular architecture, where most PCT cells appeared necrotic with a massive loss of brush borders, a marked cytoplasmic hypertrophy and vacuolization, as well as with a severe sign of nuclear pyknosis, was evidenced. Besides, most PCT and DCT cells exhibited irregular tubular arrangement and displayed cellular shedding in the lumen. Peritubular inflammatory cellular infiltration and congestion were highly detected among most renal tubules (Figure 6e,f and Figure 7e,f).

## 4. Discussion

LS, OM, FH, and ES were considered beneficial plants for a long time, but more attention has been focused on their adverse effects on human and animal health. The liver and kidneys are considered to be the main target for many compounds found in botanicals [23,35]. This study assessed the impact of LS, FH, OM, and ES on the function and structure of the liver and kidneys.

Body weight is one of the most important indicators when determining an animal’s health condition [36]. All the rats in this investigation showed typical body weight increases without any significant variations, suggesting that the plant extracts had no adverse effects on the total body weight. The relative organ weight is considered to be an indicator of toxic effect. An elevated organ-to-body weight ratio is a sign of inflammation [37]. In fact, in the present study, LS, ES, and VitE significantly increased relative liver weight. The present findings are consistent with prior work where *S. occidentalis* seed extract, also, evidenced a relative liver weight relative to the control group and induced hepatotoxicity in mice [38]. The mentioned plant extracts had varied impacts on hepatic functional markers, some of which were detrimental. On the other hand, plant extracts administration had no effect on absolute nor relative kidney weight in all experimental groups but exerted renal damage. These observations are supported by the outcomes of another study, where treatment with *S. guineense* did not statistically modify the kidneys’ weight but altered other renal functional markers [39].

Furthermore, the blood levels of ALT and AST rise following liver damage due to elevated membrane permeability, necrosis of the liver cells, and serum cytosol leakage [40]. For this reason, ALT and AST are considered to be precise indicators of liver damage, with ALT being the most sensitive liver enzyme to identify damage to liver cells [41,42]. The outcomes of the current investigation revealed that the plant extracts exhibited no significant impact on AST levels. However, ALT levels in the FH-treated group were increased. These results are in accordance with the outcomes of Sikander et al. [43], where different concentrations of *Origanum vulgare* extract did not affect ALT level, after a period of 15 days. On the other hand, the current findings that concern LS contradict a previous study, where it was found to increase ALT levels and cause hepatotoxicity similarly to what was reported in the FH group [44]. This contradiction might be due to the different doses used in the study or the larger chosen sample size. Moreover, excretory function is assessed by creatinine clearance. The renal blood flow is also evaluated using this indicator [45]. In the current study, despite the observed histological alterations, the absence of increased creatinine may indicate that the alterations may not have been severe enough to affect renal filtration. Second, creatinine is normally synthesized in the liver, stored as phosphocreatine in muscles, and filtered by the kidney [46]. Hence, the decreased levels observed in our study may result from factors not associated with renal impairment, such as impaired liver function, which has been linked to decreased creatinine production in some cases [47]. This report contradicts the study of Balgoon [48], where LS restored the normal creatinine level after a period of 8 weeks. Urea is generated by the liver in the urea cycle as a waste product of protein metabolism [49]. The primary method by which the body eliminates extra nitrogen is through the renal glomeruli’s ability to filter urea from the blood into the urine [50]. It is a sensitive biomarker that is used to evaluate the damage to renal tissue where its increase reflects nephrotoxicity [51]. In the current study, serum urea increase in the OM, ES, and VitE groups indicates that the corresponding extracts could potentially have a toxic effect on the kidneys. In fact, the histological assessment disclosed moderate–severe renal histopathological features in the OM and ES groups. Comparably, a previous study demonstrated that saffron increased blood urea nitrogen and exerted renal damage on mice neonates [52]. Interestingly, Sarwar Alam et al. [53] have previously highlighted the antioxidant and nephroprotective roles of ES, where the extract given for 7 days (dose regimen 50–200 mg/kg) restored serum urea levels and alleviated HgCl_2_-induced kidney tissues damage, which contradicts the current findings. The observed results may be attributed to the short study period, as prolonged herbal consumption has the potential to generate nephrotoxicity, in contrast to acute consumption, as previously reported [54].

Excessive reactive oxygen species (ROS) generation plays a part in the patho-physiology and etiology of numerous illnesses [55]. The excessive generation of free radicals or a lack of antioxidants including GSH and SOD results in the disruption of cellular redox balance [56]. The most prevalent intracellular thiol-based antioxidant, glutathione, plays a fundamental role in preserving cell integrity as well as acting as a major first line of defense against oxidative stress and the damaging effects of ROS on cells [57]. In the present investigation, OM extract decreased levels of kidney GSH, as confirmed by the histological findings. These results contradict previous findings where OM was found not to affect GSH in the kidney and exhibited a nephroprotective effect against prallethrin-induced damage, after 28 days of daily administration, which might be explained on the basis of the duration of the treatment [58]. On the other hand, liver GSH levels remained statistically similar to the control in all experimental groups. Polyunsaturated fatty acids in cell membranes undergo oxidative change known as lipid peroxidation, which produces a variety of breakdown products and causes oxidative stress [59]. Thus, the lipid peroxidation measurement is a crucial sign in the evaluation of antioxidant capacity. MDA, an end product following lipid peroxidation, is a widely used marker [60]. In the present investigation, liver MDA levels were increased in the ES-treated group, as similarly reported with green tea extract [61]. Kidney MDA levels were elevated in the ES and Mix groups, with the highest recorded level being in the Mix group. The pathological lesions observed in the histological study confirm the current findings, as similarly reported by Li et al. [62].

OM as well as FH and ES were found to cause mild to moderate histopathological modifications, respectively. These alterations include loss of hepatic trabecular architecture, ballooning, micro-steatosis, vacuolization, and signs of necrosis, suggesting a potential hepatotoxic effect. In fact, loss of trabecular architecture indicates structural damage, while ballooning, micro-steatosis, and vacuolization indicate fat accumulation and cellular change, related to liver damage [63]. Necrosis further denotes injury and cell death [64]. These findings are in line with earlier research where the aqueous leaf extract of *Ecliptaalba* caused comparable histopathological damage in the liver of mice [40]. OM as well as ES and Mix displayed renal histopathological features that were graded as moderate–severe and severe, respectively. These modifications included reduction in the dimensions of some renal corpuscles, tubular degeneration and necrotic signs in PCTs and DCTs, and peritubular inflammatory cellular infiltration. These outcomes suggest potential damage to different constituents of the kidneys. The reduction in renal corpuscles may be related to a decrease in the number of functional filtration units, impairing the general kidney role [65]. The tubular degeneration and necrotic signs in the PCTs and DCTs suggest impairment in the renal tubules in charge of reabsorption and secretion [66]. Furthermore, the occurrence of inflammatory cellular infiltration around the tubules could indicate an immune response to the renal damage, probably contributing to the observed histopathological changes [67]. These findings are in line with previous ones, where aristoclam, a metabolite of one of the components of Aristolochiaceae plants, caused nephrotoxicity in the form histopathological injuries in the kidneys, including disintegration of tubular epithelial cells, inflammatory cell infiltration, and fibrosis [68].

It is important to emphasize that this study has certain limitations: first, the 60-day treatment duration, which was selected based on our previous research that was necessary to assess the effect of plant extracts on spermatogenesis [24]. However, this duration induced toxicity in the liver and kidneys, as shown in the present study. Therefore, a shorter time of exposure is required to investigate the effect of these plant extracts. Another limitation includes the fixed doses of the selected folk herbs that revealed signs of hepatic and renal toxicity. Therefore, other doses can be used to assess their margins of safety. Moreover, this study did not investigate the effect of the studied plant extracts on hematological parameters and inflammatory response. Hence, they can be considered additional tests to assess the effect of these plant extracts on overall health. Additionally, the study was conducted only on male rats and not females, so further research is necessary to evaluate any gender-specific effects.

## 5. Conclusions

In conclusion, the present investigation demonstrated that the daily administration of aqueous herbal extracts via oral gavage, for 60 days, either alone or combined, generally caused liver and kidney tissue damage, reduced their antioxidant status, and affected their functional markers. OM, ES, and especially FH were revealed to cause more hepatic damage than the other plants, whereas ES and Mix were found to increase the kidneys’ level of toxicity. Therefore, it is highly recommended to not exceed the duration of 60 days during the studied plant extracts’ consumption. Additional research involving different time frames is required in order to clearly understand the onset of toxicity. Such studies would yield important information for the formulation of recommendations aimed at guaranteeing the safe application of these plants, either alone or in combination, in a variety of applications.

## Figures and Tables

**Table 1:** Table summarizing the experimental groups and treatments of rats.

Group	Number of Rats	Name	Treatment
1	4	Ctrl	Normal saline
2	4	LS	*Lepidium sativum* extract
3	4	OM	*Origanum majorana* extract
4	4	FH	*Ferula hermonis* extract
5	4	ES	*Eruca sativa* extract
6	4	Mix	Mixture of all herbs
7	4	VitE (positive control)	Vitamin E

**Table 2:** Changes in body weight (g), kidney and liver weight, and indices among experimental groups.

Experimental Groups	Body Weight (g)	Kidney Weight (g)	Kidney Index	Liver Weight (g)	Liver Index
Ctrl	348.75 ± 17.96	2.50 ± 0.29	0.74 ± 0.09	9.25 ± 0.48 ^a^	2.27 ± 0.50 ^a^
LS	378.75 ± 12.65	2.46 ± 0.28	0.65 ± 0.02	14.18 ± 0.69 ^b^	3.74 ± 0.11 ^b^
OM	368.75 ± 12.14	2.46 ± 0.10	0.66 ± 0.003	12.78 ± 1.56 ^ab^	3.44 ± 0.33 ^ab^
FH	393.75 ±8.75	2.35 ± 0.88	0.60 ± 0.03	11.78 ± 1.20 ^ab^	2.97 ± 0.25 ^ab^
ES	397.50 ±19.84	2.59 ± 0.05	0.65 ±0.04	14.61 ± 1.06 ^b^	3.66 ± 0.15 ^b^
Mix	371.25 ± 11.43	2.53 ± 0.09	0.68 ± 0.04	11.17 ± 0.24 ^ab^	3.01 ± 0.03 ^ab^
VitE	408.75 ± 11.43	2.69 ± 0.08	0.66 ± 0.01	14.89 ± 0.90 ^b^	3.64 ± 0.16 ^b^
Results of one-way ANOVA (group)	F = 2.138 *p* > 0.05	F = 0.698 *p* > 0.05	F = 1.057 *p* > 0.05	F = 4.585 *p* < 0.05	F = 4.121 *p* < 0.05

Data are means ± SEM; n = 4 rats per group. Values belonging to the same column, having distinct letters, are significantly different (*p* < 0.05). Ctrl, control group; FH, *Ferula hermonis*-treated group; OM, *Origanum majorana*-treated group; ES, *Eruca sativa*-treated group; LS, *Lepidium sativum*-treated group; Mix, plant mixture-treated group; VitE, vitamin E-treated group.

## Data Availability

The data presented in this study are available on request from the corresponding authors.

## Abbreviations

The following abbreviations are used in this manuscript:

OM	*Origanum majorana*
LS	*Lepidium sativum*
ES	*Eruca sativa*
FH	*Ferula hermonis*
PA	pyrrolizidine alkaloid
SD	Sprague Dawley
VitE	vitamin E
ALT	alanine transaminase
AST	aspartate aminotransferase
GSH	glutathione
SOD	superoxide dismutase
MDA	malondialdehyde
NBT	nitro blue tetrazolium
TBA	thiobarbituric acid
DTNB	5,5’-dithio-bis(2-nitrobenzoic acid)
H&E	hematoxylin and eosin
CREA	creatinine
PCTs	proximal convoluted tubfules
DCTs	distal convoluted tubules
BC	Bowman’s capsule
SEM	standard error of the mean
BW	body weight
